# Elevated pulmonary tuberculosis biomarker miR-423-5p plays critical role in the occurrence of active TB by inhibiting autophagosome-lysosome fusion

**DOI:** 10.1080/22221751.2019.1590129

**Published:** 2019-03-22

**Authors:** Huihui Tu, Su Yang, Tingting Jiang, Liliang Wei, Liying Shi, Changming Liu, Chong Wang, Huai Huang, Yuting Hu, Zhongliang Chen, Jing Chen, Zhongjie Li, Jicheng Li

**Affiliations:** a Institute of Cell Biology, Zhejiang University School of Medicine, Hangzhou, People’s Republic of China; b School of Medicine, South China University of Technology, Guangzhou, People’s Republic of China; c Department of Pneumology, Shaoxing Municipal Hospital, Shaoxing, People’s Republic of China; d Department of Clinical Laboratory, Zhejiang Hospital, Hangzhou, People’s Republic of China

**Keywords:** Tuberculosis, miR-423-5p, diagnostic biomarker, autophagosome-lysosome fusion; *VPS33A*

## Abstract

Rapid diagnosis of pulmonary tuberculosis is an effective measure to prevent the spread of tuberculosis. However, the grim fact is that the new, rapid, and safe methods for clinical diagnosis are lacking. Moreover, although auto-lysosome is critical in clearing *Mycobacterium tuberculosis*, the pathological signiﬁcance of microRNAs, as biomarkers of tuberculosis, in autophagosome maturation is unclear. Here, these microRNAs were investigated by Solexa sequencing and qPCR validation, and a potential diagnostic model was established by logistic regression. Besides that, the mechanism of one of the microRNAs involved in the occurrence of tuberculosis was studied. The results showed that the expression of miR-423-5p, miR-17-5p, and miR-20b-5p were signiﬁcantly increased in the serum of patients with tuberculosis. The combination of these three microRNAs established a model to diagnose tuberculosis with an accuracy of 78.18%, and an area under the curve value of 0.908. Bioinformatics analysis unveiled miR-423-5p as the most likely candidate in regulating autophagosome maturation. The up-regulation of miR-423-5p could inhibit autophagosome maturation through suppressing autophagosome–lysosome fusion in macrophages. Further investigations showed that *VPS33A* was the direct target of miR-423-5p, and the two CUGCCCCUC domains in *VPS33A* 3’-UTR were the direct regulatory sites for miR-423-5p. In addition, an inverse correlation between *VPS33A* and miR-423-5p was found in peripheral blood mononuclear cells of patients with tuberculosis. Since the inhibition of autolysosome formation plays a critical role in tuberculosis occurrence, our ﬁndings suggests that miR-423-5p could suppress autophagosome–lysosome fusion by post-transcriptional regulation of *VPS33A*, which might be important for the occurrence of active tuberculosis.

## Introduction

Pulmonary tuberculosis (TB) is a chronic respiratory infection caused by *Mycobacterium tuberculosis* (*Mtb*). According to the World Health Organization (WHO) report, there were 10.4 million new TB cases and 1.7 million deaths worldwide in 2016. TB remains the leading cause of death in infectious diseases, and the TB epidemic is more serious than previously estimated [1]. Unlike latent TB, active TB is contagious and harmful to the public [2]. So rapid diagnosis of active TB is an effective measure to prevent its spread and timely treatment [3]. However, the gold standard for diagnosing TB still depends on the old bacteriological test, which is time-consuming and with only 30% positive rate [3,4]. The Xpert MTB/RIF® test was recommended by the WHO in 2010 for TB diagnosis, because it can deliver results in 2 h. However, there is a potential risk for exposure to pathogens during this test [5]. Therefore, it is urgent to develop a rapid and safe method for TB diagnosis.

Several serological proteins have been studied for diagnosing TB [6,7]. However, the poor stability and complex operation limit the reproducibility [8] of protein detection, which further limits their clinical applications. In comparison, serum microRNAs (miRNAs) are more stable and easier to detect [9,10]. So, miRNAs with small size are potentially good disease biomarkers [11–14]. Meanwhile, miRNAs play important roles in the occurrence, development, and prognosis of diseases [15,16], as they have been predicted to regulate around 60% of human genes [17]. miRNAs affect various diseases through regulating biological processes, such as autophagy [18]. Autophagy can degrade dysfunctional or unnecessary cytoplasmic cargos, which eventually fuse with late endosomes or lysosomes [19]. Previous studies have demonstrated that autophagy can wipe out intracellular *Mtb* by autolysosomes, which is the more effective antimicrobial pattern than phagosomes [20]. The immune system and the pathogen are always in a dynamic balance [21]. Blocking the autophagic flux leads to impaired clearance of unnecessary cargos and pathogens, which can induce serious diseases such as TB [22–26]. Several studies have shown that toxic *Mtb* can inhibit autophagy activation and block autophagic flux through regulating the expression of miRNAs, thereby helping *Mtb* survival and colonization in macrophages [27–29]. In the process of autophagosome maturation, ESX-1 type VII secretion system and early secreted antigenic target of 6kD (ESAT-6) have been reported to block autophagic flux through inhibiting the *Mtb* containing phagosomes into degradable autolysosomes [30,31], which helps *Mtb* avoid being killed in immunocytes. However, the association between miRNAs and active TB regarding the autophagosome maturation process have rarely been reported. Therefore, we are interested in identifying whether there are magic-miRNAs in TB patients that can inhibit the formation of autolysosome, and whether these miRNAs are related to the occurrence of active TB.

In our study, a rapid, highly accurate, and pathogen non-contacted potential TB diagnostic model was established by screening serum miRNAs profiles with Solexa sequencing and qRT-PCR validation in a large cohort of TB patients. Furthermore, the cellular and molecular biological mechanisms of one of the diagnostic miRNAs were investigated by focusing on the autophagosome maturation process. Our study showed that miRNA regulation plays an important role in inhibiting autolysosome formation during the occurrence of active TB.

## Materials and methods

### Patients and healthy controls

The study was approved by the Medical Ethics Committee of the Faculty of Medicine Zhejiang University, China. Written informed consents were obtained from all blood donors prior to experiments.

A total of 108 untreated active TB patients (42 females, 66 males; aged 18–72) were recruited from Shaoxing Municipal Hospital and First Hospital of Jiaxing (China) between February 2014 and March 2017. 86 Healthy Controls (HCs) (36 females, 50 males; aged 21–64) were recruited from Zhejiang Hospital (China) between August 2016 and July 2017. TB patients were diagnosed relied on the diagnostic criteria of the Ministry of Health, China [32]. TB patients with hepatic, renal, blood, endocrine, metabolic and autoimmune disorders, malignant tumors, and long-term use of immune suppressive agents were excluded from the study. All patients were followed up for six months. All TB patients and HCs came from the Han population in Southeast China.

The fasting early morning blood samples were collected using 5 ml serum tubes. Serum was isolated within 4 h, by centrifuging for 10 min, under the condition of 3000 rpm and 4°C. The supernatant was transferred and stored at – 80°C before the experiments.

### Solexa sequencing

Approximately 1 μg total RNA purified from 10 TB patients or 10 HCs underwent Solexa sequencing. These RNAs were extracted with a Plasma/Serum Exosome RNA isolation Kit (Norgen, 49200). Small RNA libraries were constructed with a TruSeq Small RNA Sample Prep Kits (Illumina, RS-200). Single-end sequencing (50 bp) was performed on an illumina HiSeq2000 following previous protocol [33]. Sequences with lengths of 18–26 nucleotides were mapped to the specific species precursors in miRBase 21.0 by BLAST, to identify the miRNAs. The mapped pre-miRNAs were further BLAST with the Homo genomes to determine their locations.

### Bioinformatics analysis

The Volcano Plot of the differentially expressed serum miRNAs was performed by GraphPad Prism 5. The GO annotation was analyzed by the GO database (http://geneontology.org/). The KEGG pathway mapping was performed by the KEGG Mapper (http://www.genome.jp/kegg/mapper.html). The functional enrichment analysis was performed with DAVID (https://david.ncifcrf.gov/). The target gene prediction was performed using miRanda algorithm (http://www.microRNA.org/) and TargetScan (http://www.targetscan.org/). The interacting network was performed with Cytoscape 3.2.1.

### Cell culture and transfection

The cells of Human monocytic leukemia cell line (THP-1) were maintained in RPMI 1640 Medium (Gibco, A1049101), supplemented with 10% fetal bovine serum (Gibco, 10099141) and 0.05 mM 2-mercaptoethanol. The cell line has been tested for excluding mycoplasma contamination, and was cultured in the incubator of 5% CO_2_ and 37°C.

The stable cell line THP-1-LC3-GFP was established by transfecting pEGFP-LC3-GFP into THP-1 cells, using an Amaxa Lonza Nucleofector® Kit V (Lonza, VCA-1003). The selection pressure of geneticin was 400 ng/μl.

For miRNA or small interfering RNA (siRNA) transfection, THP-1 cells were induced with Phorbol ester (50 nM, Sigma, P1585) for 36 h, then were transfected with miRNA/siRNA (100 nM) using a Ribofect CP trans Kit (RiboBio, c10511-1) as described. A 5 μl of miRNA/siRNA was diluted into 60 μl 1 × ribo FECT™ CP Buffer (final concentration of 100 nM), and mixed gently. Then, a 6 μl of ribo FECT™ CP Reagent was added into the mixture. After incubating at room temperature for 15 min, the mixture was added to the cultured cells in a 12-well plate. After incubating for another 36 h, the transfected cells were treated with starvation or not before the tests.

### RNA extraction and qRT-PCR

PBMCs were isolated using Ficoll-Hypaque (Solarbio, P9011), and were frozen (−80°C) in Trizol at a concentration of 5 × 10^6^/ml before RNA extraction. The total miRNA of the serum was isolated using a miRcute Serum/plasma miRNA isolation Kit (Tiangen, DP503), and was reverse trancripted into cDNA by a miRcute plus miRNA first-strand cDNA synthesis Kit (Tiangen, KR211-01). The total RNA of the cells was isolated using a miRcute miRNA isolation kit (Tiangen, DP501), and was reverse trancripted into cDNA either by the Fastking RT kit (Tiangen, KR116) or by the miRcute plus miRNA first-strand cDNA synthesis kit.

SYBR green qRT-PCR assay was realized with the Lightcycler 480 SYBR Green I master (Roche, 4887352001). Each sample was run in triplicate. The expression level of miRNA was normalized with hsa-miR-16 [8]. The expression level of mRNA was normalized with β-actin. The negative control for templates was ddH_2_O. The sequences of qPCR primers are listed in Table S4.

### Western blot

The antibodies of anti-LC3 (ab48394), anti-P62 (ab91526), anti-β-actin (ab8227), and anti-rabbit IgG (Alexa 680) (ab186696) were purchased from Sigma-Aldrich. The antibodies of anti-FYCO1 (NBP1-47266), anti-VPS33A (NBP2-20872), anti-CLEC16A (NBP2-57150), and anti-CLN3 (NBP2-43782) were purchased from the Novus Corporation.

For western blot analysis, the proteins lysed from treated cells were denatured and loaded onto SDS-PAGE gels (8–15%). After electrophoresis, protein bands were transferred to a PVDF membrane. Then the PVDF membrane was blocked in the bovine serum albumin medium (5% w/v) for 1–2 h, then incubated in the primary antibody for 2–3 h and in the secondary antibody for 1 h. The specific bands were analyzed on an Odyssey CLx Infrared Imaging System. Image Studio Ver5.2 was used for densitometric analysis.

### Dual luciferase reporter assay

The sequences, whether wildtype or mutant of Homo *VPS33A* 3′-UTR (ENST00000267199) were cloned into the 3′ end of the report gene of pmirGLO Dural-luciferase plasmid (Promega, E1330), using a ClonExpress® II One Step Cloning Kit (Vazyme, C112). Mutagenesis PCR and gene recombination were performed with a Mut express® II fast mutagenesis kit V2 (Vazyme, C214). Clone and mutagenesis PCR primers are listed in Table S4.

MiRNA and plasmid were co-transfected into cells using Lipo3000 (Invitrogen, L3000001) according to the manufacturer’s instructions. A 3 μl of Lipofectamine® 3000 Reagent was diluted into 50 μl Opti-MEM® Medium. In another tube, 1 μg DNA and 5 μl miRNA (final concentration of 100 nM) were diluted into 50 μl Opti-MEM® Medium, then 2 μl P3000^TM^ Reagent was added and mixed well. Then 53 μl of diluted DNA was added into the diluted Lipofectamine® 3000 Reagent (1:1 ratio), and incubated for 5 min at room temperature. Then the mixture was added to the cultured cells, which had a density of 70–90% in a 12-well plate. Then the transfected cells were incubated for 72 h at 37°C.

Firefly and renilla luciferase luminescence were measured with a Dual-Glo® Luciferase Assay System (Promega, E2920) as described. The transfected cells were collected and transfered into a 96-well plate (75 μl/well), which was compatible with the luminometer. Then we added an equal volume of Dual-Glo® Reagent into each well and mixed, and incubated at room temperature for at least 10 min. Then the firefly luminescence was measured in a luminometer (Molecular Devices, M5). Another 75 μl volume of Dual-Glo® Stop & Glo® Reagent was added into each well and mixed, and incubated for another 10 min. Then the Renilla luminescence was measured using the luminometer. The value of firefly luciferase luminescence/Renilla luciferase luminescence represents the activity of 3′-UTR. Each sample was run in triplicate.

### Live-cell imaging

For co-localization observation, the induced macrophages from THP-1-GFP-LC3 were transfected with miRNA as described above (50 nM BA treated for 4 h as positive control), and incubated in HBSS for 4 h or not, and stained with Lyso-tracker red DND (Invitrogen, L7528) for 3 min before confocal imaging. During live imaging, the culture chamber was maintained under 37°C and 5% CO_2_. LC3-GFP dots and LAMP1-red dots were quantified with an Olympus Fluoview 3000. Image data was processed by MetaMorph 7.0.

For the DQ-BSA experiment, the induced macrophages were stained with DQ^TM^ Red BSA (10 μg/ml, Invitrogen, D12051) for 1 h in HBSS, then were cultured for another 3 h in HBSS without DQ^TM^-BSA before quantifying. Image data was processed by ImageJ.

### Electron microscopy

The induced macrophages were transfected with miRNA as described above. Cells (for 4 h HBSS treated or not) were harvested after EDTA-trypsin treatment, and washed with PBS (pH 7.4) twice. Cells were ﬁxed in 2.5% glutaraldehyde for at least 4 h at 4°C. After washing in PBS three times, cells were postﬁxed in 1% osmium tetroxide for 1 h. After washing twice in ddH_2_O, cells were ﬁxed in 2% uranylacetate for 0.5 h, then dehydrated with gradient ethanol, and embedded in Epon 812. Ultrathin sections were stained with uranylacetate and lead citrate, and were observed using a TECANL-10 electron microscope.

### Statistical analysis

The *P* value for age was tested with an Independent-samples T test, while for gender it was tested with a Chi-square test. Other clinical indexes were tested with One-Sample T Test. The molecular experiment data of two groups were tested with a T Test. The Non-parametric Mann–Whitney U test was used to compare the miRNA levels in serum or PBMCs of the two groups. The spearman correlation coefficient was performed to test the correlation of two parameters.

The relative expression levels of the miRNAs or mRNAs were calculated with the *ΔΔ*CT method [23]. Scatter diagrams and spearman correlation analysis were performed using GraphPad Prism 5. ROC analysis was performed with MedCalc 12.4.2.0. The 10-fold cross validation was performed with MATLAB.

## Results

### Case information

We designed a case–control study. The characteristics of TB patients and HCs were statistically analyzed (Table 1). There was no significant difference in age or gender between the two groups. No differences were found in Hepatitis B (HBV) infection, Human Immunodeficiency Virus (HIV) infection, and other chronic or autoimmune diseases between the two groups. Among all the patients, the sputum smear positive rate was about 70%, the sputum culture positive rate was about 59%, and the incidence rate of TB lesion on chest X-ray/CT scan was about 80%.

**Table 1.** Case information of TB patients and healthy controls.

Characteristics	Pulmonary TB			Healthy Controls		
	Solexa sequencing/training set	validation set	PBMCs extraction	Solexa sequencing/training set	validation set	PBMCs extraction
Number of cases	10	60	38	10	41	35
Age(media ± SD)	33.40 ± 12.75	37.50 ± 15.44	37.24 ± 15.10	36.80 ± 10.81	38.28 ± 10.25	38.54 ± 10.85
* P* valve of age (TB vs HCs)	0.53	0.60	0.72	0.53	0.60	0.72
Sex(female/male)	4/6	24/36	14/24	4/6	20/21	12/23
* P* valve of sex (TB vs HCs)	1	0.38	0.38	1	0.38	0.38
Sputum smear test (positive No./rate)	7/70%	42/70%	27/71.05%	NA	NA	NA
Sputum culture test (positive No./rate)	6/60%	35/58.33%	23/60.53%	NA	NA	NA
Chest X-ray/CT scan (tuberculosis lesion positive No./rate)	9/90%	46/76.67%	31/81.58%	NA	NA	NA
Hepatitis B (negative No./rate)	10/100%	60/100%	38/100%	10/100%	41/100%	35/100%
HIV (negative No./rate)	10/100%	60/100%	38/100%	10/100%	41/100%	35/100%
Other chronic inflammation or autoimmune diseases (negative No./rate)	10/100%	60/100%	38/100%	10/100%	41/100%	35/100%

The age of the cases are presented as the *mean ± SD*. HIV: Human Immunodeficiency Virus. NA: Not applicable.

### MiR-423-5p is up-regulated in the serum of TB patients and establishes a potential TB diagnostic model when combined with miR-17-5p and miR-20b-5p

**Figure 1.**
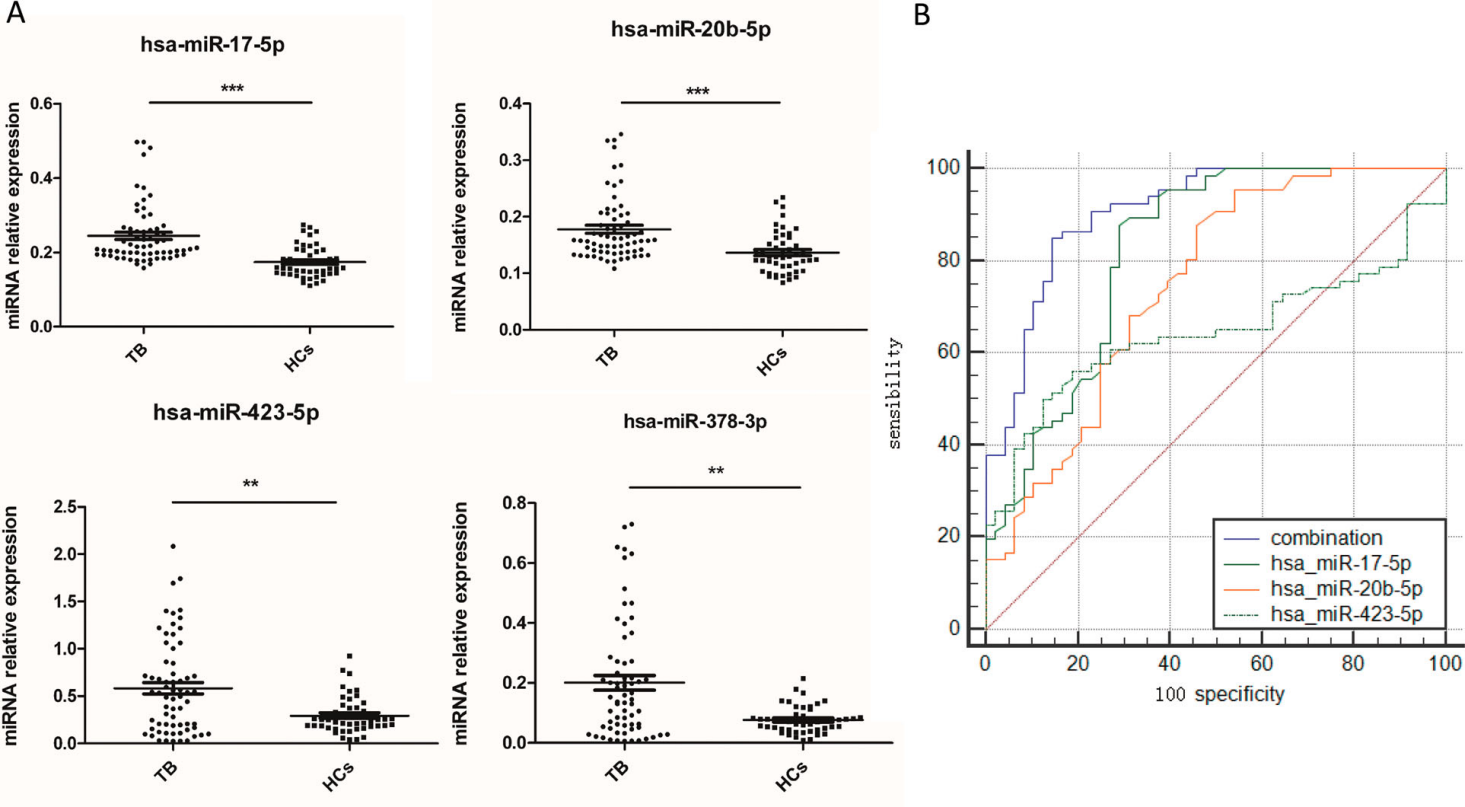
A potential TB diagnostic model was established when combined miR-423-5p, miR-17-5p and miR-20b-5p as a panel. (A) The levels of four serum miRNAs were measured by qPCR from TB patients (*N* = 70) and healthy controls (HCs, *N* = 51). Median values are shown by horizontal lines. (B) The Receiver Operating Characteristic curve of miR-17-5p, miR-20b-5p, miR-423-5p and the combined model were analyzed. N: number of subjects. *P < 0.05, ** *P *< 0.01, *** *P* < 0.001.

**Figure 2.**
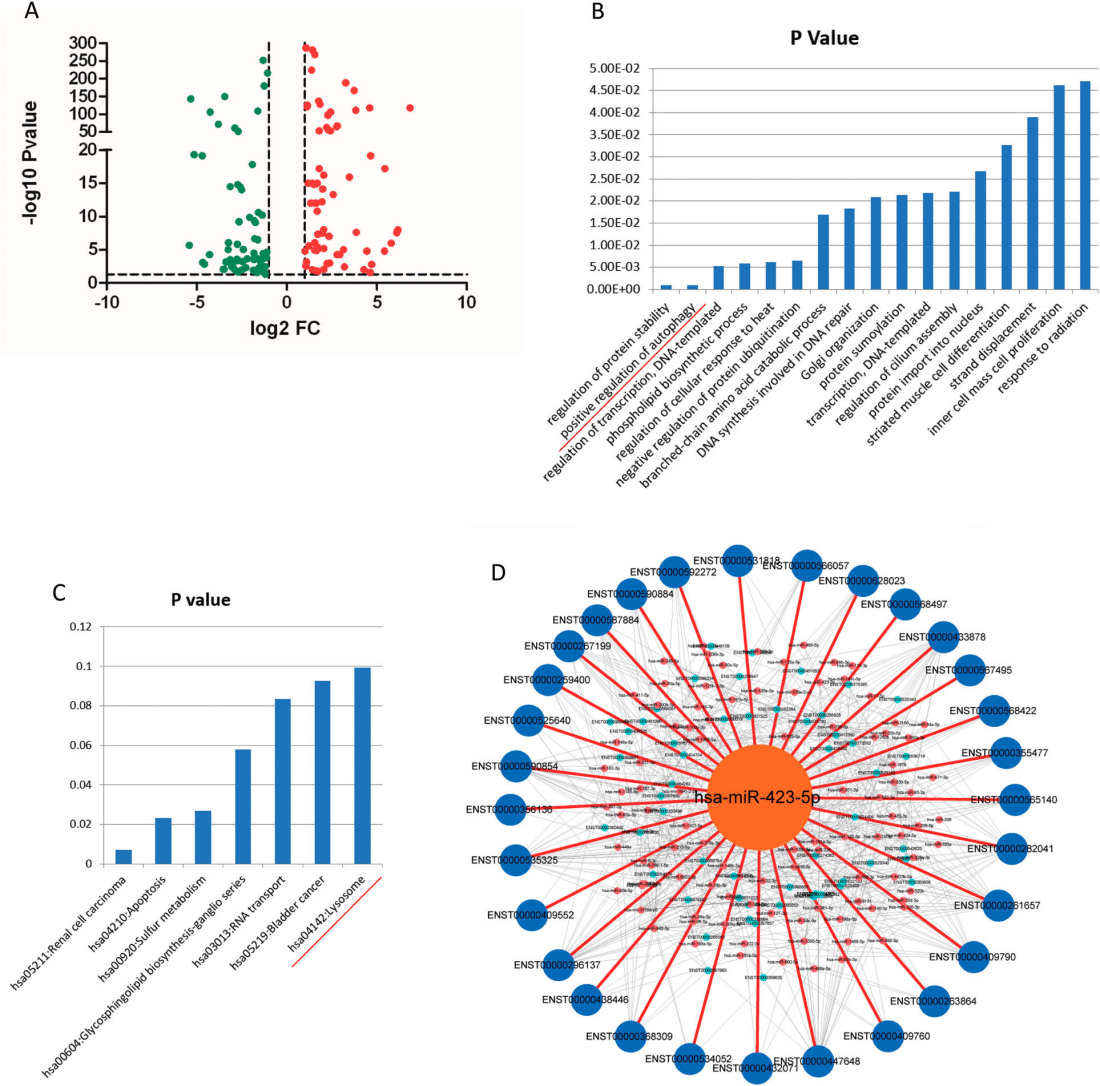
MiR-423-5p was predicted as a candidate in regulating autophagosome maturation. (A) The Volcano Plot of the differentially expressed miRNAs from TB patients and healthy controls (HCs). (B) Genes targeted by differentially expressed miRNAs were enriched in the Gene Ontology term of positive regulation of autophagy. (C) Genes targeted by differentially expressed miRNAs were enriched in the lysosome pathway. (D) All of the 30 autophagosome maturation-related genes were targeted by differentially expressed miRNAs.

We identified 181 differentially expressed serum miRNAs by Solexa sequencing between 10 TB patients and 10 HCs (fold change > 2, *P* < 0.05) (Table S1), of which 93 miRNAs were up-regulated (>2.0-fold, *P *< 0.05) and 88 miRNAs were down-regulated (<0.50-fold, *P *< 0.05) (Figure 2(A)). Nineteen miRNAs (Table S1) which met the following two criteria were validated by qRT-PCR: (1) autophagosome maturation related genes (Table S2) were the target of the miRNA; (2) the miRNA concentration was more than 10 copies in Solexa sequencing. In the training set, 10 miRNAs with fold changes (TB patients/HCs) ≥ 1.2 or ≤ 0.8, and *P *< 0.05 were selected for further analysis. In the validation set, these 10 miRNAs were further measured in a large cohort of 60 TB patients and 41 HCs. The results showed that the expression levels of miR-17-5p (*P < *0.001), miR-20b-5p (*P < *0.001), miR-378a-3p (*P < *0.01), and miR-423-5p (*P < *0.01) were significantly up-regulated in TB patients (Figure 1(A)), and had the same trend as the Solexa results.

We performed Receiver Operating Characteristic (ROC) analysis to evaluate the sensitivity and specificity of the miRNAs. The area under the curves (AUC) of miR-17-5p, miR-20b-5p, miR-378a-3p, and miR-423-5p were 0.822, 0.747, 0.677, and 0.644, respectively. We established a diagnostic model using forward stepwise multivariate regression. MiR-378a-3p was excluded from the model because the significance of its contribution to the model was 0.667 (Table S3). When the other three miRNAs served as a panel, the sensitivity, specificity, and the AUC increased to 84.8%, 85.4%, and 0.908, respectively (Figure 1(B)). Further, we tested the diagnostic accuracy of this model using 10-fold cross validation [33]. The results indicated that it could discriminate TB patients from HCs with an accuracy of 78.18% (83.93% sensitivity, 71.44% specificity).

### Bioinformatics analysis indicates miR-423-5p as a candidate in regulating autophagosome maturation

Genes targeted by the differentially expressed miRNAs were enriched in 17 Gene Ontology (GO) terms, including positive regulation of autophagy (*P *< 0.001, Figure 2(B)). Genes targeted by the differentially expressed miRNAs were enriched in 7 Kyoto Encyclopedia of Genes and Genomes (KEGG) pathways, including the lysosomal pathway (Figure 2(C)). These results indicated that the differentially expressed miRNAs in TB patients have correlation with autophagy and lysosome.

Further, 30 autophagosome maturation related genes were downloaded from the GO database (Table S2). We predicted the relationships between up-regulated miRNAs and autophagosome maturation genes, and between up-regulated miRNAs and genes that positively regulated autophagosome maturation, using miRanda algorithm with cutoff scores of ≥ 140 and free energy ≤ −20.0 kCal/Mol [34]. The relationships between down-regulated miRNAs and genes that negatively regulated autophagosome maturation were predicted using the same approach. Surprisingly, 30 autophagosome maturation genes were targeted by all of the differentially expressed miRNAs (Figure 2(D)), which indicated that there was a strong correlation between the fluctuating miRNAs and autophagosome maturation. Further analysis found that miR-423-5p targeted the most transcripts (Figure 2(D)), indicating that miR-423-5p may play an important role in the autophagosome maturation process.

### MiR-423-5p inhibits autophagosome maturation by blocking autophagosome-lysosome fusion in macrophages

**Figure 3.**
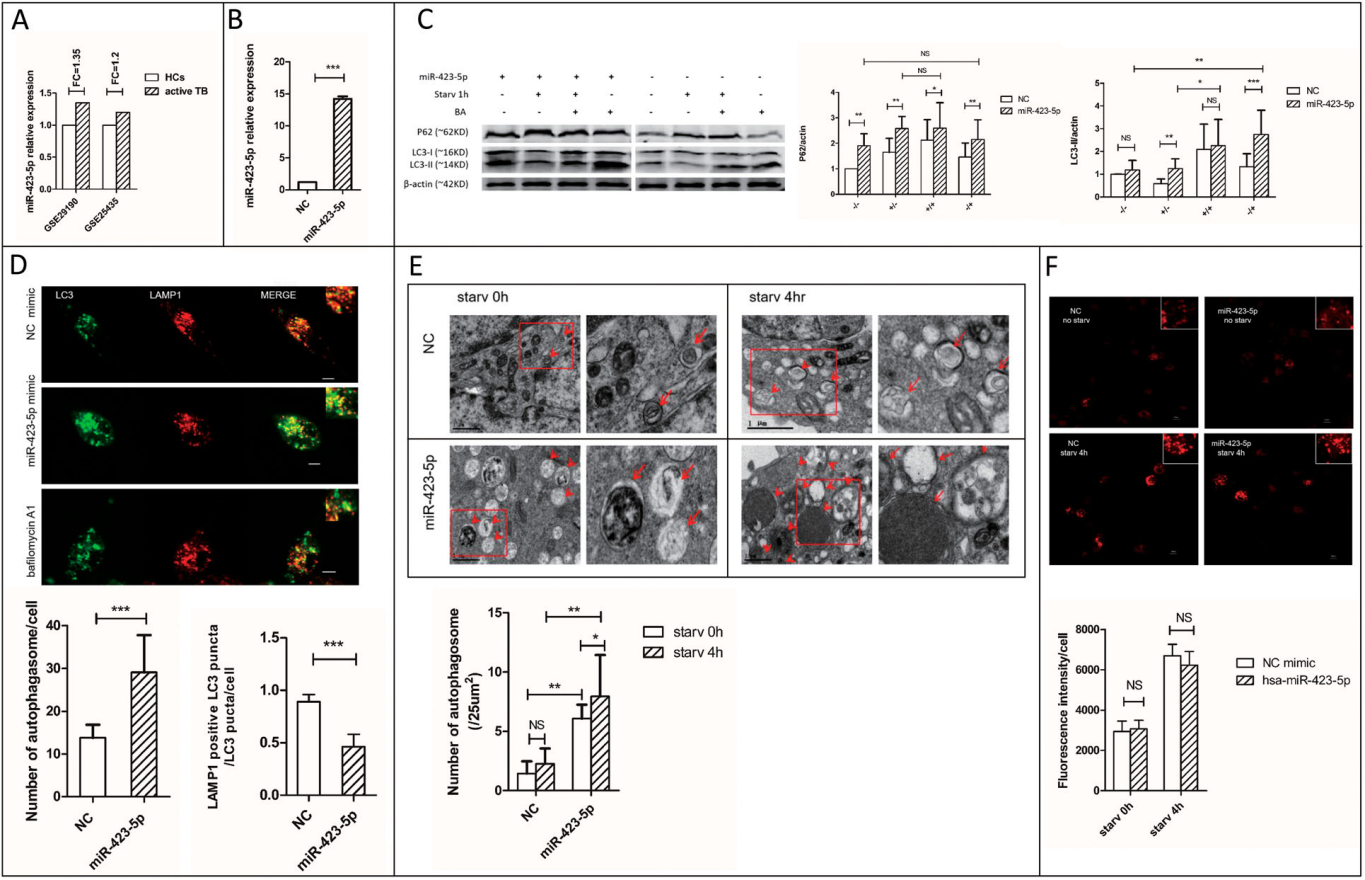
MiR-423-5p inhibits autophagosome maturation by suppressing autophagosome-lysosome fusion. (A) The expression ratio of mir-423-5p in the PBMCs of TB patients/healthy controls (HCs) from GSE29190 and GSE25435. (B) The levels of miR-423-5p in the cells transfected with miR-423-5p mimic or NC mimic. (C) The protein levels of P62 and LC3-II in the cells transfected with miR-423-5p mimic or NC mimic were tested by western blot. Target protein was normalized with β-actin. −/−: no starvation/no BA, +/−: 1 h starvation/no BA, +/+: 1 h starvation/50nM BA, −/+: no starvation/50nM BA. The experiments repeated more than three times. (D) The number of LC3-GFP dots and the co-localization of LC3-GFP puncta with lysosomes (lysotracker red DND) in the macrophages treated with miR-423-5p mimic or NC mimic were analyzed. More than 20 macrophages from three independent experiments were analyzed. Scale bars: 5μm. (E) Autophagosomes in the macrophages treated with miR-423-5p mimic or NC mimic were observed by electron microscopy. Autophagosomes are indicated by arrows. The number of autophagosomes from an accumulated area of >200 μm^2^ (>20 cells) was statistically analyzed. Scale bars: 1μm. (F) The lysosomal proteolytic activity of the macrophages treated with miR-423-5p mimic or NC mimic was analyzed by DQ-BSA. The fluorescent intensity from at least 40 cells was quantified. Scale bars: 10 μm. All statistical data, except (A) are presented as *mean ± SD*, and error bars indicate the *SD*. NC: negative control. *P < 0.05, ** *P *< 0.01, *** *P* < 0.001.

We analyzed the transcriptomes (GSE29190, GSE25435) of peripheral blood mononuclear cells (PBMCs) from TB patients and HCs. The expression of miR-423-5p was up-regulated in the PBMCs of TB patients (Figure 3(A)).

Sequestosome-1 (SQSTM1/P62) serves as a link between microtubule-associated protein 1 light chain 3 beta (LC3) and ubiquitinated substrates [35]. SQSTM1-bound poly-ubiquitinated proteins can be incorporated into the completed autophagosome and degraded in autolysosomes,so SQSTM1/P62 can be used as a protein marker of autophagic degradation [36]. SQSTM1/P62 in combination with LC3-II is widely used to monitor autophagic flux [37]. In the starvation groups, the expression of P62 (1.6-fold, *P *< 0.01) and LC3-II (2.1-fold, *P *< 0.01) increased in the macrophages treated with miR-423-5p mimic, when compared with negative control (NC). The accumulation of P62 (1.7-fold, *P *< 0.01) and LC3-II (1.2-fold) in the miR-423-5p mimic treated cells even occurred under growing condition (Figure 3(C)). Moreover, the accumulation of P62 showed stronger response to miR-423-5p mimic than to bafilomycin A1 (BA, positive control), whether the cells were starvated or not. The P62 expression in the miR-423-5p mimic treated cells did not increase further by additional treatment with BA. These results suggested that in macrophages, the autophagic ﬂux was blocked in the autophagosome maturation process after the miR-423-5p mimic treatment.

We established a stable cell line THP-1-LC3-GFP to observe autophagosomes. In the miR-423-5p mimic and starvation treated macrophages, the co-localization ratio of GFP-LC3 puncta with lysosomes decreased about 2-fold (*P *< 0.001), and the number of autophagosomes increased significantly (*P *< 0.001), when compared with the NC mimic and starvation treated group. This phenomenon happened even under growing conditions (Figure 3(D)). Electron microscopic observation showed significant accumulation of autophagosomes (initial autophagic vacuoles, AVi) in the miR-423-5p mimic treated cells, whether they were in the starvation group (3.5-fold, *P *< 0.01) or not (4.2-fold, *P *< 0.01) (Figure 3(E)). The diameter of autophagosomes in the miR-423-5p mimic treated cells easily reached 1.5 μm, while the largest was 1 μm in the NC mimic treated cells. These results suggested that autophagosome-lysosome fusion was blocked after the miR-423-5p mimic treatment.

The protein level of SQSTM1/p62 also increased when the proteasome was inhibited [38]. So, we further assessed the lysosomal degradation capacity using DQ^TM^ Red BSA. The results showed there was no significant difference in lysosomal proteolytic activity between the cells transfected with miR-423-5p mimic and NC mimic, whether in the starvation group or not (Figure 3(F)). In conclusion, miR-423-5p inhibited autophagosome maturation by blocking autophagosome-lysosome fusion, rather than by interfering with autolysosome degradation.

### VPS33A is negatively regulated by miR-423-5p

**Figure 4.**
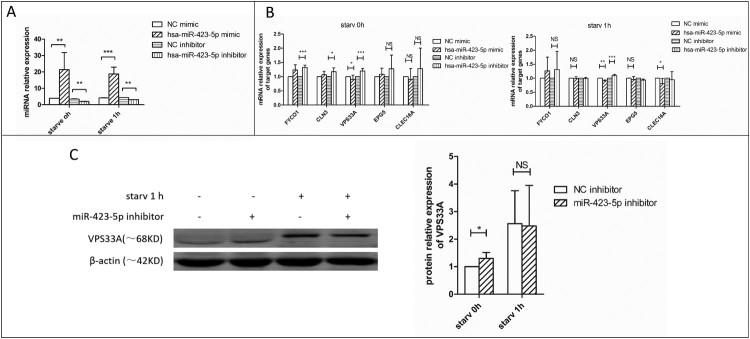
The mRNA and protein expression levels of VPS33A were measured. (A) The relative expression levels of miR-423-5p in the cells transfected with mimic (NC or miR-423-5p) or inhibitor (NC or miR-423-5p). Target miRNA was normalized with hsa-miR-16. (B) The mRNA levels of the five target genes in the cells transfected with mimic (NC or miR-423-5p) or inhibitor (NC or miR-423-5p) were analyzed, whether the cells were starvated or not. (C) The protein levels of VPS33A in the macrophages transfected with inhibitor (NC or miR-423-5p) and those treated with starvation (0 h or 1 h) were analyzed. The statistical results were obtained from more than three independent experiments. Target genes were normalized with β-actin. Data are presented as *mean ± SD*, and error bars indicate the *SD*. NC: negative control. * *P *< 0.05, ** *P *< 0.01, *** *P* < 0.001.

**Figure 5.**
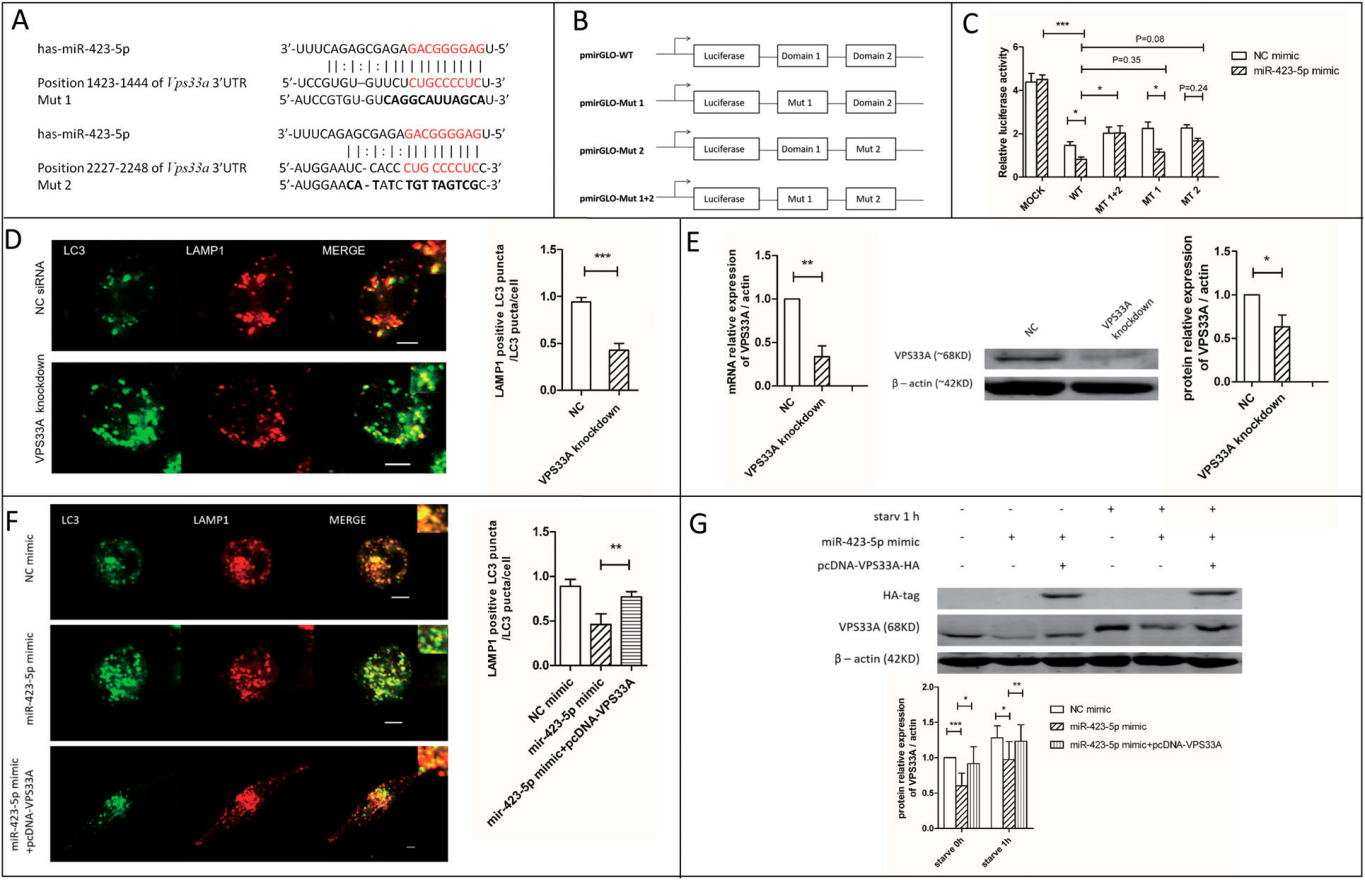
*VPS33A* was the direct target of miR-423-5p. (A) The location of the two miR-423-5p binding domains. Interaction domains are indicated by red font. Mutant sequences are indicated by bold font. (B) The structure diagram of the constructed plasmids. (C) A 2 × 4 (factor of miRNA: NC mimic and miR-423-5p mimic, 100 nM; factor of luciferase reporter plasmid: pmirGLO vector (mock), pmirGLO-WT, pmirGLO-MT1, pmirGLO-MT2 and pmirGLO-MT (1 + 2)) orthogonal experiment for Dual-Glo-Luciferase-Assay was performed. (D) The co-localization of LC3-GFP puncta with lysosomes in the macrophages transfected with VPS33A specific siRNA (negative siRNA as NC) was analyzed. More than 20 cells from three independent experiments were analyzed. Scale bars: 5 μm. (E) The expression levels of VPS33A in the macrophages transfected with *VPS33A* specific siRNA or NC siRNA were analyzed. (F) The co-localization of LC3-GFP puncta with lysosomes in the macrophages transfected with NC mimic, miR-423-5p mimic, or miR-423-5p mimic + pcDNA-VPS33A were analyzed. More than 20 cells from three independent experiments were analyzed. Scale bars: 5 μm. (G) The protein levels of VPS33A in the macrophages transfected with NC mimic, miR-423-5p mimic, or miR-423-5p mimic + pcDNA-VPS33A were analyzed by western blot. The statistical results were obtained from more than three independent experiments. Target genes were normalized with β-actin. Data are presented as *mean ± SD*, and Error bars indicate the *SD*. NC: negative control. *P < 0.05, ** *P* < 0.01, *** *P* < 0.001.

FYVE and coiled-coil domain-containing protein 1 (*FYCO1*)*,* Battenin (*CLN3*)*,* Ectopic P granules protein 5 homolog (*EPG5*)*,* Vacuolar protein sorting-associated protein 33A (*VPS33A*)*,* and C-type lectin domain family 16 member A (*CLEC16A*) were the target genes of miR-423-5p predicted by miRanda and TargetScan [39]. We quantified the messenger RNA (mRNA) levels of the five genes (Figure 4(B)). The results showed that the mRNA level of *VPS33A* decreased significantly in the miR-423-5p mimic treated macrophages, whether in the starvation group (0.9-fold, *P *< 0.01) or not (0.8-fold, *P *< 0.05). On the contrary, it increased in the cells treated with miR-423-5p inhibitor, regardless of its starvation group membership (1.15-fold, *P *< 0.001) or not (1.2-fold, *P *< 0.001). Further, we quantified the protein levels of FYCO1, CLN3, VPS33A and CLEC16A. Only VPS33A showed a 30-40% decrease (*P *< 0.05) in the miR-423-5p mimic treated macrophages (Figure 5(G)), while no significant changes were observed in CLEC16A, FYCO1 and CLN3, whether in the starvation group or not. Inversely, the protein level of VPS33A increased (1.3-fold, *P *< 0.05) in the miR-423-5p inhibitor transfected macrophages (Figure 4(C)). The results above indicated that VPS33A was negatively regulated by miR-423-5p.

### *VPS33A* is the direct target of miR-423-5p

It was predicted by miRanda and TargetScan that, there were two miR-423-5p binding domains CUGCCCCUC located in the position 1423–1444 and 2227–2248 of *VPS33A* 3′-UTR (Figure 5(A)). We subcloned the wild-type sequence of Homo *VPS33A* 3′-UTR into pmirGLO, to produce pmirGLO-WT. Mutants (Mut 1, Mut 2 and Mut 1 + 2) were cloned as described in Figure 5(A,B).

In the Dual-Glo-Luciferase-Assay, the relative luciferase activity was decreased (5.5-fold, *P *< 0.001) in the miR-423-5p mimic + pmirGLO-WT transfected cells, when compared with the miR-423-5p mimic + pmirGLO group. The relative luciferase activity was increased significantly (2.5-fold, *P *< 0.05) in the miR-423-5p mimic + pmirGLO-MT1 + 2 transfected cells, compared with the miR-423-5p mimic + pmirGLO-WT group. It was also increased in the cells transfected with miR-423-5p mimic + pmirGLO-MT1 or miR-423-5p mimic + pmirGLO-MT2, but was less than the miR-423-5p mimic + pmirGLO-MT1 + 2 group (Figure 5(C)). These results indicated that miR-423-5p was sensitive to the change of domain 1423–1444 or domain 2227–2248 in *VPS33A* 3′-UTR.

The relative luciferase activity decreased 45% (*P *< 0.05) in the miR-423-5p mimic + pmirGLO-WT transfected cells, compared with the NC mimic + pmirGLO-WT group. When the cells were transfected with pmirGLO-MT1 + 2, the Dual-Glo-Luciferase-Assay System was insensitive to the increase of miR-423-5p concentration. But when the cells were transfected with pmirGLO-MT1 or pmirGLO-MT2, the miR-423-5p mimic brought the decrease of relative luciferase activity (Figure 5(C)). These results indicated that both domains 1423–1444 and 2227–2248 were sensitive to the change of miR-423-5p concentration, and they performed a superimposed effect.

There was a 60% decrease (*P *< 0.001) in the co-localization rate of GFP-LC3 puncta with lysosomes in starved macrophages, after *VPS33A* was knocked-down (Figure 5(D,E)). Additionally, the co-localization rate increased to 1.6 fold (*P *< 0.01, Figure 5(F)) and the protein level of VPS33A increased to 1.3-1.5 fold (*P *< 0.05, Figure 5(G)) in the miR-423-5p mimic + pcDNA-VPS33A treated group, compared with the miR-423-5p mimic group. It suggested that over-expression of VPS33A could partially rescue the inhibiting effect of miR-423-5p in autophagosome-lysosome fusion. Taken together, these results indicated that miR-423-5p could inhibit autophagosome-lysosome fusion by post-transcriptionally inhibiting the expression of *VPS33A* through direct interaction with the CUGCCCCUC domains of the 3′UTR.

### *VPS33A* expression is reduced and inversely correlated with miR-423-5p in the PBMCs of TB patients

**Figure 6.**
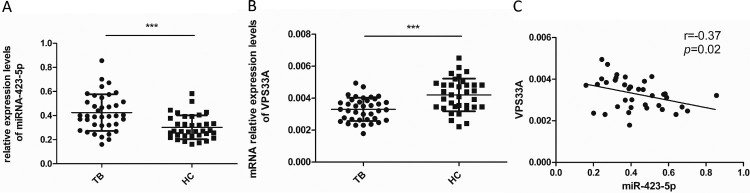
Spearman correlation coefficients revealed an inverse correlation between the mRNA levels of *VPS33A* and miR-423-5p in the PBMCs of TB patients. (A) The levels of miR-423-5p were measured by qPCR in the PBMCs of TB patients (*N* = 38) and HCs (*N* = 35). (B) The mRNA levels of *VPS33A* were measured by qPCR in the PBMCs of TB patients (*N* = 38) and HCs (*N* = 35). (C) Spearman correlation coefficient between the mRNA levels of *VPS33A* and miR-423-5p in the PBMCs of TB patients was calculated. Median values are shown by horizontal lines. *N*: number of subjects. TB: pulmonary tuberculosis. HCs: healthy controls. * *P *< 0.05, ** *P *< 0.01, *** *P* < 0.001.

We isolated the PBMCs from TB patients (*N* = 38) and HCs (*N* = 35), and assessed the mRNA levels of *VPS33A* and miR-423-5p in PBMCs. Consistent with the results from GSE29190 and GSE25435 (Figure 3(A)), the miR-423-5p levels significantly increased in PBMCs from TB patients (1.4-fold, *P *< 0.001, Figure 6(A)), compared with HCs. Conversely, the mRNA levels of *VPS33A* decreased by 20% (*P *< 0.001, Figure 6(B)) in PBMCs from TB patients. Further, in the PBMCs of TB patients, a negative correlation between the mRNA levels of *VPS33A* and miR-423-5p was found (*r* = −0.37, *P *= 0.02; Figure 6(C)). This data suggested that the expression of *VPS33A* was suppressed in PBMCs of TB patients, which may be negatively regulated by the increased miR-423-5p.

### Clinical data analysis reveals a correlation between miR-423-5p and IgM, and between miR-423-5p and B-factor

Autophagy is an important immune pattern in macrophages and plays a connection role between immune regulatory factors and the immune protection against intracellular pathogens [40]. To understand the overall clinical immune status of TB patients, and its correlation with miR-423-5p which has inhibitory effects on autophagy, we collected and analyzed the relevant parameters of immune function from 70 TB patients (the same as miRNAs validation). The results between TB patients and HCs showed significant differences in neutrophil percentage (*P *< 0.001), lymphocyte percentage (*P *< 0.001), monocyte percentage (*P *= 0.007), basophil percentage (*P *= 0.001), Immunoglobulin A (IgA, *P *= 0.001), Immunoglobulin M (IgM, *P *= 0.001), Complement C3 (C3, *P *< 0.001), Complement C4 (C4, *P *= 0.001), B-factor (*P *< 0.001), and C-reactive protein (CRP, *P *= 0.001)(Table 2). Moderate correlations between miR-423-5p and IgM (*r *= 0.47, *P *< 0.01), and between miR-423-5p and B-factor (*r *= 0.3, *P *< 0.05), were found in the serum of TB patients.

**Table 2.** Clinical parameters of immune function for TB patients and healthy controls.

	TB (*N* = 70)	HC (*N* = 51)	*P* Value (TB vs Control)	*r* (with serum miR-423-5p)
hemameba (×10^9^/L)	6.67 ± 2.27	3.50–9.50	0.52	−0.02
blood platelet (×10^9^/L)	277. 03 ± 90.00	125–350	0.001**	0.026
percentage of neutrophil (%)	62.73 ± 11.68	40–75	<0.001***	−0.15
percentage of lymphocyte (%)	25.90 ± 10.68	20–50	<0.001***	0.19
percentage of monocyte (%)	7.24 ± 2.23	3–10	0.007**	−0.10
percentage of eosinophils (%)	3.94 ± 2.41	0.40–8	0.502	0.01
percentage of basophil (%)	0.31 ± 0.28	0–1	0.001**	0.18
IgG (g/L)	13.86 ± 4.64	11.50–14.22	0.102	−0.16
IgA (g/L)	3.00 ± 1.20	1.70–3.25	0.001**	−0.13
IgM (g/L)	1.20 ± 0.53	0.73–1.17	0.001**	0.47(*P *< 0.01)
C3 (g/L)	1.18 ± 0.20	0.83–1.77	<0.001***	0.10
C4 (mg/L)	276.82 ± 101.92	100–400	0.001**	0.07
B-factor (g/L)	0.36 ± 0.07	0.10–0.50	<0.001***	0.30(*P *< 0.05)
CRP (mg/L)	13.93 ± 21.28	0–8.20	0.001**	−0.11

The clinical data of TB patients are presented as *mean ± SD*. The clinical data for HCs are presented as reference range. TB: pulmonary tuberculosis. HC: healthy control. N: number of subjects. *r*: spearman correlation coefficient. IgG: Immunoglobulin G. IgA: Immunoglobulin A. IgM: Immunoglobulin M. C3: Complement C3. C4: Complement C4. CRP: C-reactive protein.

*P < 0.05, **P < 0.01, ***P < 0.001.

## Discussion

The current clinical diagnostic methods for TB have the disadvantages of low positive rate and time consuming procedure, which seriously affects its early diagnosis and timely treatment. In our previous study of early diagnostic biomarkers of TB we considered the accuracy and safety of the methods [8,41], but lacked of in-depth study for the pathological significance of the biomarkers. Inhibition of autophagosome-lysosome fusion in macrophages plays critical role in the obstacle of clearing *Mtb* [20] and in the occurrence of TB [28]. But the role of miRNAs in inhibiting autophagosome-lysosome fusion has hardly been documented. In this study, a rapid, highly accurate, and pathogen non-contacted miRNA diagnostic model was established for TB by combining three miRNAs. Moreover, the up-regulated biomarker miR-423-5p was found playing an important role in inhibiting autophagosome-lysosome fusion in macrophages through mediating VPS33A.

Several proteins have been investigated as potential biomarkers for TB [6,7,42]. However, the disadvantages of protein, including poor stability, complex operation [8] and the lack of high-throughput multiscan spectrum limit its development as a potential biomarker. Relatively speaking, miRNAs not only have the characteristics of short sequence, difficult degradation [43], simple operation, and rapid detection, but they also can meet the requirements of clinical automation and high-throughput. Serum is the common sample in clinical practice, and there is small risk of infection in operating the serum of TB patients. Therefore, serum miRNAs may serve as good biomarkers for the diagnosis of active TB. In our study, the AUC of the model reached 0.908, and the 10-fold cross validation showed a 78.18% predictive accuracy, suggesting a good clinical value of the model in distinguishing active TB from HCs. Zhang *et al*. [8] have established a miRNA diagnostic model for active TB (AUC > 0.9), but they did not test the model accuracy with 10-fold cross validation or with double-blind experiment, which affects the assessment of its clinical value. The sensitivity of our model reached 83.93%, which is much higher than the Xpert test (about 70%) [5] and the gold standard bacteriological test (about 30%) [3,4].

MiR-423-5p is a reliable diagnostic biomarker for active TB. Before the experiment, we have ruled out the TB patients with other diseases, to ensure that the results were only affected by *Mtb* infection. Besides in the serum of TB patients, the up-regulation of miR-423-5p also occurred in the PBMCs which is the main source of macrophages for clearing *Mtb*. Additionally, Zhu *et al*. have reported that there was no difference in serum miR-423-5p level among the lung cancer, benign pulmonary disease, and HCs [44]. In another set of data from our laboratory, the expression of serum miR-423-5p was called-back in the TB patients after six months of treatment (Supplementary Table S5), which indicated that the up-regulation of miR-423-5p was related to the occurrence of TB.

Although the molecular mechanism of anti-*Mtb* in macrophages/monocytes remains unclear, growing evidence has shown that toxic *Mtb* could modulate immune response (by inhibiting the NF-Κb pathway [45] or decreasing IFN-γ responsiveness [46]) through altering host miRNA expression. In this study, we found that the up-regulated miR-423-5p in TB patients can inhibit the formation of auto-lysosomes in the macrophages through inhibiting its target gene *VPS33A*. Zhang *et al*. and Romagnoli *et al*. have shown that *Mtb* could modulate protein expression, thereby blocking autophagic flux through inhibiting autophagosome-lysosome fusion [30,31]. Our findings support the hypothesis that *Mtb* also attempts to inhibit auto-lysosome formation in TB patients through regulating other response factors (i.e. host miRNAs). VPS33A plays a key role in mediating autophagosomes-lysosomes fusion [47]. In the miR-423-5p mimic transfected macrophages, the inhibition of autophagosome-lysosome fusion was similar to that of VPS33A knockdown [48,49]. miR-423-5p could negatively regulate VPS33A in the PBMCs of TB patients, which indicated that VPS33A plays a central regulatory role in the inhibition of downstream autophagosome-lysosome fusion by miR-423-5p. In the studies of the upstream of miR-423-5p regulation, transcription factor nuclear factor erythroid-derived 2 has been indicated to induce the miR-423-5p expression in HepG2 cells [50]. In summary, the direct regulation by proteins and the regulation by miRNAs are different approaches which lead to the same destination, i.e. *Mtb* could inhibit the normal fusion of autophagosomes and lysosomes in the immune cells. This is similar to the ability of active *Mtb* to inhibit lysosomes from participating in phagocytosis, thus avoiding to be cleared [51]. Granuloma formation is a milestone of the occurrence of active TB [52–54]. Blocking lysosomes to participate in various degradation processes could help *Mtb* colonization in host macrophages and the formation of granuloma [52]. Coincidentally, Liu *et al*. [55] have reported that HBV fought against the immune clearance through suppressing autophagosome maturation. Yang *et al*. [56] have reported that in the chronic airway infection of *Pseudomonas aeruginosa*, autophagy was also inhibited by the pathogen to resist the host’s defense. So, it seems that inhibiting lysosomes from participating in autophagosome maturation is a common escape mechanism of chronic infectious bacteria or virus to avoid being cleared by the host’s immune system and to colonize in the host’s immune cells.

In the starvation groups, VPS33A was not increased in the miR-423-5p inhibitor group. This may be associated with the increased basal level of VPS33A protein, or with the translational regulation or other post-transcriptional regulation of VPS33A under starvation condition. Moreover, the shift of VPS33A was delayed after starvation. The shift delay was also observed in oxygen and glucose deprived cells, but it did not take place when autophagy was induced by Torin 1(data not shown). It suggested that the shift delay of VPS33A might be specific to nutrient deprivation. These are worthy to be studied further. Given the significance of autolysosome in clearing *Mtb* [20] and the strict control for only *Mtb* infection when enrolling patients, the increased miR-423-5p has important pathological significance in the occurrence of active TB through inhibiting the fusion of autophagosome and lysosome. However, a bacterial clearance test could better demonstrate the direct relationship between the increased miR-423-5p and the obstacle of removing *Mtb*. It is well known that when subjected to pathogens, autophagy plays a role in the innate immune function in macrophages [57]. In this study, the clinical data showed a moderate correlation between miR-423-5p and the immune-related indicators such as IgM and B – factor. This suggested that IgM and B-factor may be related to the inhibition of auto-lysosome formation through miR-423-5p, or that miR-423-5p may also play a role in other immune pathways. This is worthy to be investgated in the future work.

In summary, the present study suggests that miR-423-5p as an early diagnostic biomarker for TB can inhibit the fusion of autophagosomes and lysosomes, which may play a key role in the occurrence of active TB. The results further clarify the fact that the model established in this study has important diagnostic and pathological significance in active TB diagnosis. From the perspective of miRNAs, our study provides a new paradigm for studying *Mtb* escape and the occurrence of active TB caused by the anti-microbial defects in the lysosomal pathway.

## Supplementary Material

Supplemental Material

## References

[1] World Health Organization Global tuberculosis report of 2017; 2017.

[2] Barry CE, Boshoff HI, Dartois V, et al. The spectrum of latent tuberculosis: rethinking the biology and intervention strategies. Nat Rev Microbiol. 2009;7:845–855. doi: 10.1038/nrmicro2236 19855401 PMC4144869

[3] Pm S. Tuberculosis: a new vision for the 21st century. Kekkaku. 2009;84:721–726. 19999594

[4] Heydari AA, Movahhede Danesh MR, Ghazvini K. Urine PCR evaluation to diagnose pulmonary tuberculosis. Jundishapur J Microbiol. 2014;7:e9311. doi: 10.5812/jjm.9311 PMC4138658 25147688

[5] Lawn SD, Mwaba P, Bates M, et al. Advances in tuberculosis diagnostics: the Xpert MTB/RIF assay and future prospects for a point-of-care test. Lancet Infect Dis. 2013;13:349–361. doi: 10.1016/S1473-3099(13)70008-2 23531388 PMC4844338

[6] Liu J, Jiang T, Wei L, et al. The discovery and identification of a candidate proteomic biomarker of active tuberculosis. BMC Infect Dis. 2013;13:506. doi: 10.1186/1471-2334-13-506 24168695 PMC3870977

[7] Xu D-D, Deng D-F, Li X, et al. Discovery and identification of serum potential biomarkers for pulmonary tuberculosis using iTRAQ-coupled two-dimensional LC-MS/MS. Proteomics. 2014;14:322–331. doi: 10.1002/pmic.201300383 24339194

[8] Zhang X, Guo J, Fan S, et al. Screening and identification of six serum microRNAs as novel potential combination biomarkers for pulmonary tuberculosis diagnosis. Plos One. 2013;8:e81076. doi: 10.1371/journal.pone.0081076 24349033 PMC3857778

[9] Gilad S, Meiri E, Yogev Y, et al. Serum MicroRNAs are promising novel biomarkers. Plos One. 2008;3:e3148. doi: 10.1371/journal.pone.0003148 PMC2519789 18773077

[10] Mitchell PS, Parkin RK, Kroh EM, et al. Circulating microRNAs as stable blood-based markers for cancer detection. P Natl Acad Sci USA. 2008;105:10513–10518. doi: 10.1073/pnas.0804549105 PMC2492472 18663219

[11] Kong L, Zhu J, Han W, et al. Significance of serum microRNAs in pre-diabetes and newly diagnosed type 2 diabetes: a clinical study. Acta Diabetol. 2011;48:61–69. doi: 10.1007/s00592-010-0226-0 20857148

[12] Goren Y KM, Zafrir B, Tabak S, et al. Serum levels of microRNAs in patients with heart failure. Eur J Heart Fail. 2012:14; 147–154. doi: 10.1093/eurjhf/hfr155 22120965

[13] Shi W, Du J, Qi Y, et al. Aberrant expression of serum miRNAs in schizophrenia. J Psychiatr Res. 2012;46:198–204. doi: 10.1016/j.jpsychires.2011.09.010 22094284

[14] Ji F, Yang B, Peng X, et al. Circulating microRNAs in hepatitis B virus-infected patients. J. Viral Hepat.. 2011;18:e242–e251. doi: 10.1111/j.1365-2893.2011.01443.x 21692939

[15] Chen L, Zhou Y, Sun Q, et al Regulation of autophagy by MiRNAs and their emerging roles in tumorigenesis and cancer treatment. International Review of Cell & Molecular Biology. 2017;334:1–26. doi: 10.1016/bs.ircmb.2017.03.003 28838537

[16] Matthew A, Sermersheim KHP, Kristyn Gumpper TM, et al. MicroRNA regulation of autophagy in cardiovascular disease. Frontiers in Bioscience. 2017;22:48–65. doi: 10.2741/4471 PMC5349319 27814601

[17] Di Leva G, Garofalo M, Croce CM. MicroRNAs in cancer. Annu Rev Pathol: Mech Dis. 2014;9:287–314. doi: 10.1146/annurev-pathol-012513-104715 PMC4009396 24079833

[18] Frankel LB, Lund AH. MicroRNA regulation of autophagy. Carcinogenesis. 2012;33:2018–2025. doi: 10.1093/carcin/bgs266 22902544

[19] Yang Z, Klionsky DJ. Mammalian autophagy: core molecular machinery and signaling regulation. Curr Opin Cell Biol. 2010;22:124–131. doi: 10.1016/j.ceb.2009.11.014 20034776 PMC2854249

[20] Ponpuak M, Davis AS, Roberts EA, et al. Delivery of cytosolic components by autophagic adaptor protein p62 Endows autophagosomes with unique antimicrobial properties. Immunity. 2010;32:329–341. doi: 10.1016/j.immuni.2010.02.009 20206555 PMC2846977

[21] Ehlers S, Schaible UE. The granuloma in tuberculosis: Dynamics of a host–pathogen collusion. Front Immunol. 2013;3:411. doi: 10.3389/fimmu.2012.00411 PMC3538277 23308075

[22] Deretic V. Autophagy in tuberculosis. Cold Spring Harb Perspect Med. 2014;4:a018481–a018481. doi: 10.1101/cshperspect.a018481 25167980 PMC4208715

[23] Kroemer G. Autophagy: a druggable process that is deregulated in aging and human disease. J Clin Invest. 2015;125:1–4. doi: 10.1172/JCI78652 25654544 PMC4382251

[24] White E. The role for autophagy in cancer. J Clin Invest. 2015;125:42–46. doi: 10.1172/JCI73941 25654549 PMC4382247

[25] Menzies FM, Fleming A, Rubinsztein DC. Compromised autophagy and neurodegenerative diseases. Nat Rev Neurosci. 2015;16:345–357. doi: 10.1038/nrn3961 25991442

[26] Lavandero S, Chiong M, Rothermel BA, et al. Autophagy in cardiovascular biology. J Clin Invest. 2015;125:55–64. doi: 10.1172/JCI73943 25654551 PMC4382263

[27] Kim JK, Yuk J-M, Kim SY, et al. MicroRNA-125a inhibits autophagy activation and antimicrobial responses during mycobacterial infection. The Journal of Immunology. 2015;194:5355–5365. doi: 10.4049/jimmunol.1402557 25917095

[28] Kim JK, Lee H-M, Park K-S, et al. MIR144* inhibits antimicrobial responses against mycobacterium tuberculosis in human monocytes and macrophages by targeting the autophagy protein DRAM2. Autophagy. 2016;13:423–441. doi: 10.1080/15548627.2016.1241922 27764573 PMC5324854

[29] Holla S, Kurowska-Stolarska M, Bayry J, et al. Selective inhibition of IFNG-induced autophagy by Mir155- and Mir31-responsive WNT5A and SHH signaling. Autophagy. 2014;10:311–330. doi: 10.4161/auto.27225 24343269 PMC5396086

[30] Romagnoli A, et al. ESX-1 dependent impairment of autophagic flux by mycobacterium tuberculosis in human dendritic cells. Autophagy. 2012;8:1357–1370. doi: 10.4161/auto.20881 22885411 PMC3442882

[31] Zhang L, Zhang H, Zhao Y, et al. Effects of mycobacterium tuberculosis ESAT-6/CFP-10 fusion protein on the autophagy function of mouse macrophages. DNA Cell Biol. 2012;31:171–179. doi: 10.1089/dna.2011.1290 21740189

[32] Ministry of Health of the People's Republic of China Diagnostic criteria for pulmonary tuberculosis.

[33] Wang C, Liu C-M, Wei L-L, et al. A group of novel serum diagnostic biomarkers for multidrug-resistant tuberculosis by iTRAQ-2D LC-MS/MS and solexa sequencing. Int J Biol Sci. 2016;12:246–256. doi: 10.7150/ijbs.13805 26884721 PMC4737680

[34] Enright AJ, John B, Gaul U, et al. MicroRNA targets in drosophila. Genome Biol. 2003;5:RI. doi: 10.1186/gb-2003-5-1-r1 PMC395733 14709173

[35] Bjorkoy G, Lamark T, Brech A, et al. P62/SQSTM1 forms protein aggregates degraded by autophagy and has a protective effect on huntingtin-induced cell death. J Cell Biol. 2005;171:603–614. doi: 10.1083/jcb.200507002 16286508 PMC2171557

[36] Mizushima N, Yoshimori T. How to interpret LC3 immunoblotting. Autophagy. 2007;3:542–545. doi: 10.4161/auto.4600 17611390

[37] Klionsky DJ, Abdalla FC, Abeliovich H. Guidelines for the use and interpretation of assays for monitoring autophagy. autophagy. 2012;8:445–544. doi: 10.4161/auto.19496 22966490 PMC3404883

[38] Bardag-Gorce F, Francis T, Nan L, et al. Modifications in P62 occur due to proteasome inhibition in alcoholic liver disease. Life Sci. 2005;77:2594–2602. doi: 10.1016/j.lfs.2005.04.020 15964033

[39] Lewis BP, Shih I, Jones-Rhoades MW, et al. Prediction of mammalian MicroRNA targets. cell. 2003;115:787–798. doi: 10.1016/S0092-8674(03)01018-3 14697198

[40] Gutierrez MG, Master SS, Singh SB, et al. Autophagy is a defense mechanism inhibiting BCG and mycobacterium tuberculosis survival in infected macrophages. Cell. 2004;119:753–766. doi: 10.1016/j.cell.2004.11.038 15607973

[41] Wang C, Yang S, Liu C-M, et al. Screening and identification of four serum miRNAs as novel potential biomarkers for cured pulmonary tuberculosis. Tuberculosis. 2018;108:26–34. doi: 10.1016/j.tube.2017.08.010 29523324

[42] Zhou J. Early diagnosis of pulmonary tuberculosis using serum biomarkers. Proteomics. 2015;15:6–7. doi: 10.1002/pmic.201400532 25431312

[43] van Rooij E, Purcell AL, Levin AA. Developing microRNA therapeutics. Circ Res. 2012;110:496–507. doi: 10.1161/CIRCRESAHA.111.247916 22302756

[44] Zhu Y, Li T, Chen G, et al. Identification of a serum microRNA expression signature for detection of lung cancer, involving miR-23b, miR-221, miR-148b and miR-423-3p. Lung Cancer. 2017;114:6–11. doi: 10.1016/j.lungcan.2017.10.002 29173767

[45] Kumar M, Sahu SK, Kumar R, et al. MicroRNA let-7 modulates the immune response to mycobacterium tuberculosis infection via control of A20, an inhibitor of the NF-κB pathway. Cell Host Microbe. 2015;17:345–356. doi: 10.1016/j.chom.2015.01.007 25683052

[46] Ni B, Rajaram MVS, Lafuse WP, et al. Mycobacterium tuberculosis decreases human macrophage IFN-γ responsiveness through miR-132 and miR-26a. The Journal of Immunology. 2014;193:4537–4547. doi: 10.4049/jimmunol.1400124 25252958

[47] Zhen Y, Li W. Impairment of autophagosome-lysosome fusion in the buff mutant mice with the VPS33A^D251E^ mutation. Autophagy. 2015;11:1608–1622. doi: 10.1080/15548627.2015.1072669 26259518 PMC4590608

[48] Jiang P, Nishimura T, Sakamaki Y, et al. The HOPS complex mediates autophagosome-lysosome fusion through interaction with syntaxin 17. Mol Biol Cell. 2014;25:1327–1337. doi: 10.1091/mbc.e13-08-0447 24554770 PMC3982997

[49] Guo X, Tu L, Gumper I, et al. Involvement of Vps33a in the fusion of uroplakin-degrading multivesicular bodies with lysosomes. Traffic. 2009;10:1350–1361. doi: 10.1111/j.1600-0854.2009.00950.x 19566896 PMC4494113

[50] Yang W, Wang J, Chen Z, et al. NFE2 Induces miR-423-5p to promote gluconeogenesis and hyperglycemia by repressing the hepatic FAM3A-ATP-Akt pathway. Diabetes. 2017;66:1819–1832. doi: 10.2337/db16-1172 28411267

[51] Daniel L, Clemens MAH. Characterization of the mycobacterium tuberculosis phagosome and evidence that phagosomal maturation is inhibited. J Exp Med. 1995;181: 257–270. doi: 10.1084/jem.181.1.257 7807006 PMC2191842

[52] Ramakrishnan L. Revisiting the role of the granuloma in tuberculosis. Nat Rev Immunol. 2012;12:352–366. doi: 10.1038/nri3211 22517424

[53] Weiss G, Schaible UE. Macrophage defense mechanisms against intracellular bacteria. Immunol Rev. 2015;264:182–203. doi: 10.1111/imr.12266 25703560 PMC4368383

[54] Giosue S, Casarini M, Ameglio F, et al. Aerosolized interferon-alpha treatment in patients with multi-drug-resistant pulmonary tuberculosis. Eur Cytokine Netw. 2000;11:99–103. 10705306

[55] Liu B, Fang M, Hu Y, et al. Hepatitis B virus X protein inhibits autophagic degradation by impairing lysosomal maturation. Autophagy. 2013;10:416–430. doi: 10.4161/auto.27286 24401568 PMC4077881

[56] Yang Z-S, Ma L-Q, Zhu K, et al. Pseudomonastoxin pyocyanin triggers autophagy: Implications for pathoadaptive mutations. Autophagy. 2016;12:1015–1028. doi: 10.1080/15548627.2016.1170256 27159636 PMC4922443

[57] Deretic V. Autophagy in innate and adaptive immunity. Trends Immunol. 2005;26:523–528. doi: 10.1016/j.it.2005.08.003 16099218

